# A facile fabrication of porous fluoro-polymer with excellent mechanical properties based on high internal phase emulsion templating using PLA as co-stabilizer†

**DOI:** 10.1039/c9ra08226c

**Published:** 2019-12-06

**Authors:** Yongkang Wang, Umair Azhar, Jinxuan He, Huiying Chen, Jianzhi Zhao, Ai-Min Pang, Bing Geng

**Affiliations:** Shandong Provincial Key Laboratory of Fluorine Chemistry and Chemical Materials, School of Chemistry and Chemical Engineering, University of Jinan Jinan 250022 China chm_gengb@ujn.edu.cn; Science and Technology on Aerospace Chemical Power Laboratory Xiangyang Hubei 441003 China hw800008@hotmail.com

## Abstract

The stability of fluoro-high internal phase emulsion (fluoro-HIPE) systems and fluoro-polyHIPEs’ mechanical strength require further improvement to meet the requirements of future applications. In this study, we used polylactic acid (PLA) as a co-stabilizer to improve the stability of the fluoro-polyHIPE. The effects of concentration and molecular weight of PLA on the pores of the fluoro-polyHIPEs were investigated. The addition of PLA produced a porous material with narrower void size distributions, higher specific surface areas and enhanced mechanical properties compared to the fluoro-polyHIPE material without the additive. The resulting fluoro-polyHIPE showed smaller pore sizes (void diameters ranged from 1–3 μm) and an improved hydrophobic nature (contact angle can reach to 148.6°). The crush strength and Young's modulus values can reach 4.42 and 74.07 MPa, respectively, at a PLA addition of 25 wt% (oil phase composition), representing increases of 246% and 650% over fluoro-polyHIPE without PLA addition. The fluoro-poly-HIPE demonstrated excellent mechanical properties compared to many engineering foams, such as melamine, polystyrene, and even graphite foams. Improvements in the performance of porous fluoropolymer materials will be beneficial for many applications, such as chemical adsorption and separation, *etc.*

## Abstract 2

Effect of PLA on the stability of fluorinated-HIPE and size tuning of the resultant fluoro-polyHIPE with enhanced mechanical properties.

## Graphical abstract

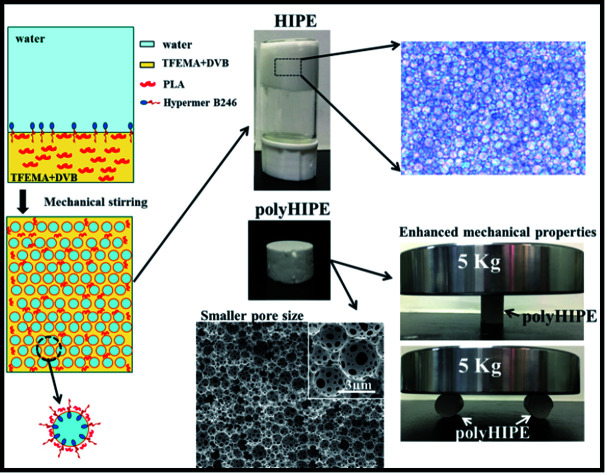

## 1. Introduction

Porous polymer foams or monoliths play an important role in a wide range of applications such as rapid oil/water separation,^1^ supporting materials for growth of tissues,^2^ catalyst attachment,^3^ controlled release matrices,^4^ separation membranes,^5^ heavy metal ion collectors,^6^ as well as other uses.^7^ Recently, a great deal of research has been carried out into highly porous polymer materials prepared using high internal phase emulsion (HIPE) templating methods.

HIPE templates serve as a pattern to produce polymer materials with a porous structure. Monomers (oil) are used as the external phase and water as the internal phase and the volume fraction of the internal phase may range from 0.74–0.99 with respect to the total emulsion volume.^8^ Candidate monomers for HIPE include styrene, acrylate, divinylbenzene, glycidyl methacrylate and so on.^9,10^ Recently, the introduction of fluoro-monomers into the polyHIPE system has shown that polymer monoliths can be prepared with the advantages of *e.g.* chemical/oxidative stability, thermal/environmental stability and excellent surface properties.^11,12^

For the HIPE system, the styrene-, acrylate- or fluoro-monomers used as the external phase must remain stable when mixed with the aqueous internal phase.^13^ A surfactant can be used to control the dispersed droplets and create voids and pore throats after polymerization. Williams^14^ demonstrated that styrene containing porous material could be prepared *via* the emulsion template method using Span 80 surfactant. Monte^15^ reported that surfactant Span 60 could be used to stabilize and prepare styrene and divinyl benzene (DVB) based emulsions.

Other workers have used co-surfactants to facilitate emulsion stability. Liu *et al.*^16^ reported a facile method to improve the stability of methyl methacrylate (MMA) HIPE using sorbitan monooleate (Span 80) as the surfactant with long-chain co-surfactant compounds added to the oil phase, such as hexadecane and hydroxylterminated polybutadiene (HTPB).

Although Zhang *et al.* stabilized the emulsion containing less than 50% fluoro-monomer with Span 80,^17^ the unique properties of fluoro-monomers can require the use of specialized, *i.e.* Hypermer-B246, rather than traditional surfactants, such as CTAB (cationic surfactant), Tween-80, Span 60, to stabilize the fluorinated emulsions.^1,18^ We also synthesized a cationic fluorosurfactant diblock copolymer (PDMAEMA-*b*-PHFBA) to prepare stable high performance fluoropolymer foams.^19^ Similarly, we prepared and used humic acid modified iron oxide nanoparticles as a co-stabilizer to enhance the stability of fluoroHIPE.^18^ Although fluoro-containing HIPEs are available in this field of research, further effort is required to improve the stability of fluorinated emulsion foams.

Currently, the pore size of the polymer materials based on HIPE templates is much larger than 10 μm, resulting in a low specific surface area and poor mechanical properties. This is significant since the mechanical performance of the resulting macroporous polymers determines their eventual applications. To a certain degree, the pore size of polyHIPE foams affects its mechanical properties. Thomas^20^ found that the compression properties of macroporous polymers with smaller average pore diameters were higher than those produced from foams with larger pore diameters.

Considerable research into the reduction in pore size of porous materials has been carried out. Since smaller cells are produced by smaller emulsion droplets, these are found in more stable emulsions. Instability of the parent emulsion is caused by two main mechanisms, droplet coalescence, and Ostwald ripening. These mechanisms lead to a coarsening of the emulsion and an increase in droplet size. There are several routes to decrease the pore size of polyHIPEs, although all have limitations. Microemulsion methods using a co-emulsifier have been tried. Luo *et al.* reported a miniemulsion template method to prepare a polymeric monolith with sub-micron pore structure: although the pore size could be reduced to the sub-micron level, the internal phase volume was relatively low (typically 40–60%), and it was difficult to meet the 74% internal phase volume requirement of our high internal phase emulsion.^21,22^

Several factors greatly influence the stability of HIPE. Among these, the nature of the continuous phase, temperature,^23^ the presence of salts in the aqueous phase, energy input during preparation,^24^ surfactant concentration, nature and volume ratios of emulsion phases,^25,26^ are the more useful factors determining HIPE kinetic stability and morphology control (*e.g.* pore size and wall thickness). Barbetta *et al.* reported that pore size could be adjusted by changing the monomer type; polyHIPEs prepared from 4-vinylbenzyl chloride (VBC) and divinylbenzene (DVB) had smaller average void diameters than those prepared from styrene and DVB and the pore size could be reduced to 5 μm by regulating the VBC content.^27^ Zhu *et al.* stated the effect of temperature on either stability of HIPE-templates or morphologies of the corresponding polyHIPEs.^23^ Zhang reported that the internal phase volume fraction affected the pore size; the pore size could be reduced to 33 μm by decreasing the internal phase volume at the expense of porosity.^28^ Cameron found that pore size could be reduced from 15 to 5 μm by changing the amount of DVB crosslinking agent although the subsequent increase in DVB weakened the performance of the host monomer.^29^ Zhang *et al.* and Chen *et al.* both reported that increasing the concentration of an aqueous solution of NaCl gave a 10 fold reduction in void diameter (from circa 30–50 μm) in the resulting polymer.^30^ However, the latter method had only a small effect on the pore size and was less effective in the fluorinated emulsion.

Hydrodynamic effects, such as viscosity of the continuous phase and interfacial tension are also important factors determining droplet coalescence and hence emulsion stability. Rezanavaz and Fee showed that increasing the average molecular weight of the crosslinking agent, *e.g.* by adding further polyethyleneglycol dimethacrylate units, increased the viscosity of the oil phase during prepared HIPE, resulting in reduced droplet coalescence and enhanced mechanical properties of the final polyHIPE.^10^ García *et al.* showed that the use of a higher viscosity deep-eutectic solvent as an alternative internal phase was also beneficial for emulsion stability.^15^ Cameron *et al.* also reported that the introduction of PLA into the continuous phase increased the viscosity of the emulsion.^31^

In this study, we selected PLA as a co-stabilizer to improve the stability of the fluoro-polyHIPE by increasing the viscosity of the continuous phase while decreasing the interfacial tension. The effects of concentration and molecular weight of PLA on the pore morphology of fluoro-polyHIPEs were thoroughly investigated. Our experiment shows polylactic acid has good compatibility with fluoromonomers. High internal phase emulsions (HIPEs) containing polylactic acid and the enhanced mechanical properties of the resulting polyHIPE materials are prepared. This type of porous fluorinated-foams has excellent oleophilicity and hydrophobicity, with water contact angles (WCA) up to 148.4° (nearly superhydrophobic) and possessed enhanced mechanical properties.

## 2. Experimental

### 2.1 Materials

d,l-Lactide (dimer of lactic acid) was ordered from Jinan Daigang Biological Engineering Co., Ltd. (Jinan, China); 2,2,2-trifuoroethyl methacrylate (TFEMA, hydrophobic monomer) was from Harbin Xeogia Fluorine–Silicon Chemical CO., Ltd. (Harbin, China); DVB (technical grade, 80%), azobisisobutryonitrile (AIBN), calcium chloride dihydrate (CaCl_2_–2H_2_O), Span 80 (sorbitanmonooleate), Span 85 (sorbitantrioleate), Span 60 (sorbitan stearate) were all from Sigma-Aldrich (USA); Tween-20 (polyoxyethylene (20) sorbaitan monolaurate), Tween-80 (polyoxyethylene sorbitanmonooleate), Sudan III dyes were provided by Sinopharm Chemical Reagent Co., Ltd (Shanghai, China); non-ionic surfactants Hypermer-B246 and Hypermer A70 were from Croda (USA); toluene and xylene (>97%) was supplied by Tianjin Fuyu Fine Chemical Company (China). The nonionic surfactant Hypermer-B246 is a polyhydroxystearic acid/polyethylene oxide/polyhydroxystearic acid ABA block copolymer.^32–35^ TFEMA and DVB were purified by passing through a column of basic alumina to remove the inhibitor. Technical grade AIBN was recrystallized from methanol and dried under reduced pressure for 12 h to yield AIBN (98%). Deionized water was used in all experiments. All other materials were used as received.

### 2.2 Preparation of hydroxy-terminated PLA

Ring-opening polymerization was used to synthesize hydroxy-terminated PLA (PLA–OH) using stannous octoate as the catalyst.^36^d-Lactide (15.0 g) was dissolved in dry xylene (35 mL) with stirring overnight at room temperature under nitrogen (positive pressure). Anhydrous benzyl alcohol (150 μL) and tin(II) 2-ethylhexanoate (150 μL) were then added and the mixture sealed and stirred for 24 h at 140 °C. When cool, the resultant polymer was precipitated from cold methanol and then washed sequentially with methanol and hexane followed by drying under reduced pressure at 40 °C for 48 h (yield, 13.2 g, 90%). The characterization of the product is shown in ESI Fig. S1.†

### 2.3 Synthesis of fluorinated porous materials

**Scheme 1:**
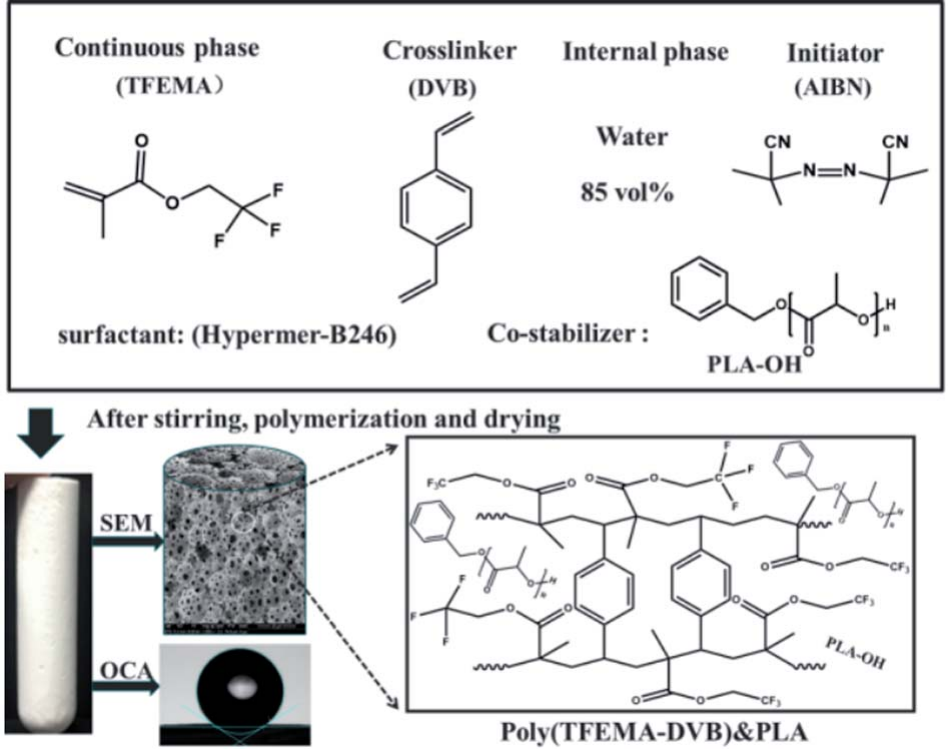
Reaction scheme of preparation of polyHIPE (TFEMA–DVB)&PLA.

A series of water-in-oil HIPEs were prepared using different surfactants at various concentrations of PLA–OH. A typical water-in-oil HIPE prepared with Hypermer B246 and 85 wt% water internal phase (see sample A2 in Table 1) was synthesized as follows: TFEMA (1.5010 g) and DVB (1.5000 g) were mixed in a glass vial. PLA–OH (0.1511 g, 5 wt%) and Hypermer-B246 (0.1502 g, 5 wt% to oil phase) were added to the oil phase mixture which was ultra-sonicated (Hypation Branson Digital Sonifer) for 10 min at room temperature to effect the complete dissolution of PLA–OH and Hypermer-B246. When the mixture had cooled to room temperature, AIBN (0.03 g, 1 wt% to oil phase) was added and the emulsion was poured into a 50 mL three-necked round-bottom flask equipped with an overhead controllable speed agitator. Aqueous calcium chloride (0.2 M, 17 g) was added dropwise with continuous mechanical stirring (500 RPM). When thickening of the emulsion was observed the speed of the agitator was decreased to 400 rpm; the speed was then increased to 500 rpm when addition of the aqueous phase was complete. The prepared emulsion was transferred to a centrifuge tube and incubated at 70 °C for 24 h to polymerize. When the emulsion was very viscous, centrifugation was necessary before polymerization. Simple centrifugation did not destroy the stability of the emulsion; the material obtained was denser with a reduction in large air bubbles because of the viscosity of the emulsion during the filling process. The reaction scheme for the preparation of a representative polyHIPEs from TFEMA and DVB with PLA as co-stabilizer is depicted in Scheme 1.

**Table 1:** Composition of high internal phase emulsion and several factors of macroporous polymers including foam average void diameter (*D*), throat diameter (*d*), specific surface area (SSA) and foam density (FD)

Samples^a^	Hypermer-B246^b^ (wt%)	PLA^b^ (wt%)	*D* (μm)	*d* (μm)	SSA (m^2^ g^−1^)	FD (g cm^−3^)
A1	5%	0%	45.24	4.73	7.24	0.1979
A2	5%	5%	9.13	1.85	10.00	0.1885
A3	5%	10%	7.95	1.82	13.22	0.1829
A4	5%	20%	5.10	0.90	15.45	0.2148
A5	5%	25%	3.07	0.47	20.80	0.2003

a For all the samples, the continuous phase of the emulsion consists of a 1 : 1 mixture of TFEMA and DVB, internal phase fraction is 85 wt% with respect to the total emulsion.

b With respect to the oil phase containing TFEMA and DVB.

## 3. Characterization

Number average molecular weight and polydispersity index (PDI) of PLA–OH (3 mL L^−1^ in THF) was determined by gel permeation chromatography (GPC) at 25 °C using a 1500 series pump and Refractive Index Detector (Waters, USA). THF was used as a mobile phase at a flow rate of 2 mL min^−1^. The molecular structures were characterized using: Fourier transform infrared spectroscopy (FTIR) from 500 to 4000 cm^−1^ at a resolution of 4 cm^−1^ using an Equinox 55 FTIR, (Brucker, USA); ^1^H NMR spectra were recorded in CDCl_3_ at room temperature using an Advance III 400 MHz nuclear magnetic resonance spectrometer (Bruker, USA). The size and the shape of HIPE droplets were observed using an Eclipse LV100POL optical microscope (Nikon, Japan). The optical micrographs were used to evaluate the droplet size distribution by counting ≥100 droplets per sample using Nano measurer software. The microstructure of porous fluoropolymers was studied by imaging fracture surfaces using a S-2500 scanning electron microscope (SEM, Hitachi Seiki Ltd., Japan). Prior to SEM, approximately 0.5 cm^3^ of each sample was fixed onto an aluminum stub using a carbon sticker and gold coated at 20 mA for 90 s using a Scan Coat Six SEM Sputter Coater (Edwards Ltd., Crawley, UK). Images of the fractured surfaces were taken from the top, middle and bottom sections to account for the variations in pore morphology from droplet coalescence and sedimentation. Pore and pore throat dimensions were analyzed using Nano Measurer 1.2 software. Specific surface areas of porous fluoropolymers were determined by nitrogen adsorption isotherm using the Brunauer–Emmett–Teller (BET) model using a TriStar II 3020 (Micromeritics, USA) surface area analyzer. Contaminants were removed *via* a “Degassing” step, prior to gas adsorption, where approximately 150 mg of each sample was heated to 200 °C in glass sample cells for 8 h. Porosities of the samples were calculated by the liquid displacement test. Hydrophobicity of porous fluoropolymers was measured using an optical contact angle instrument/drop shape analyzer (Data Physics Co., Germany) at room temperature. Reported values were the mean of five replicate measurements. The adsorption capacity of polyHIPE monolith foams was carried out using hexane, methanol, cyclohexane, petroleum ether, dichloromethane, acetone, toluene, ethyl acetate and dimethyl sulfoxide as target oils: polyHIPE monolith foam (0.15 g, cylindrical shape measuring approximately 13 mm in diameter × 12 mm in height) was immersed in an oil/water mixture and the oil intake capacity *k* was calculated as follows (eqn (1)),

1 *k* = (*m*_1_ − *m*_0_)/*m*_0_

where *m*_0_ is mass of monolith before oil adsorption; *m*_1_ is mass of monolith after oil adsorption. Three replicates were performed for each sample.

A Lloyd universal testing machine was used to measure the mechanical properties of cylindrical shaped test specimens (10 mm × 10 mm), in compression mode at a speed of 2 mm min^−1^ using a 5000 N load cell, according to ASTM D822. The Young's modulus of the samples was determined from the slope of the initial linear region of the stress–strain curves, while the maximum crush strength was obtained from the maximum compressive stress sustained before fracture. The reported values of compression stress and Young's modulus were determined from five measurements. Continuous phase viscosity was measured using an NDJ-5S digital viscometer (Shanghai Jinghai Instrument Co., Ltd., China) at 20 ± 0.01 °C; the uncertainty of the viscosity determination was 0.1%. Densities of the polyHIPEs were determined from weight and volume measurements. Interfacial tension at the oil–water interface was measured by the lifting ring method using a JK99B automatic tension meter (Shanghai Zhongchen Digital Technology Equipment Co., Ltd., China): PLA–OH or surfactant was dissolved in the oil phase, the aqueous phase was added carefully and allowed to stand at the room temperature until separation of the two layers was complete.

## 4. Result and discussion

### 4.1 Stability of HIPE and droplet size distributions with and without PLA

During the preparation of porous materials by the HIPE template method, the stability of the emulsion is directly related to the morphology and properties of the polyHIPE. Emulsions with poor stability will collide resulting in de-emulsification on standing or at elevated temperature. Hence it is difficult to obtain a complete and well-formed porous material.

The preparation of stable fluorinated emulsions is particularly difficult due to the limitations associated with available surfactants.^1,18,19^ A series of surfactants were carefully selected to stabilize water/oil (w/o) emulsions using fluorinated acrylate as a continuous phase monomer. HIPEs were then prepared using these conventional surfactants, *i.e.* Tween 20, Tween 80, Span 60, Span 80, Span 85, A70 and Hypermer B246.

**Fig. 1:**
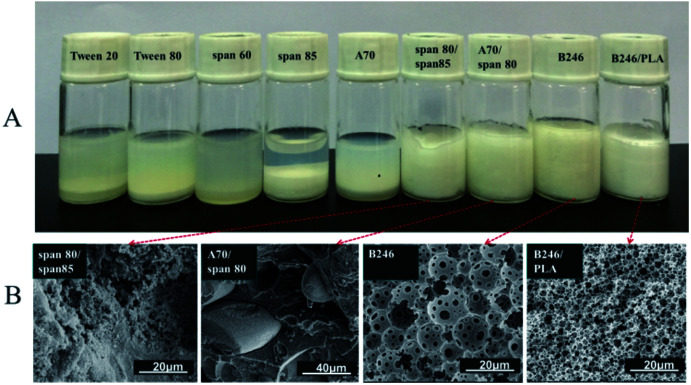
(A) Stability of w/o emulsions stabilized by different surfactants after 1 hour at room temperature and (B) corresponding SEM photographs after polymerization of emulsion.

Fig. 1A shows the images of emulsions prepared with the different surfactants at 10 wt% after standing for 1 hour at room temperature. Clear bilayer phase separation had occurred with Tween 20, Tween 80, Span 60, Span 85, A70 which were unable to stabilize the emulsions. The composite surfactants (*i.e.* Span 80/Span 85, 1 : 1 and Span 80/A70, 1 : 1) showed some stability in the emulsions but failed to form porous materials after polymerization (see Fig. 1B). Hypermer B246 has proved to be effective at stabilizing such emulsions and producing porous materials after polymerization. However, this system was not suitable for the production of porous materials with smaller pore size because of its limited stability. To overcome this limitation, a series of experiments were conducted using PLA as a co-emulsifier.

We prepared two sets of HIPE having the same surfactant concentration and aqueous phase fraction: one set contained added PLA while the second set did not. The images given in ESI Fig. S2† showed that bilayer phase separation had occurred in the HIPE prepared without PLA after standing for 5 days at room temperature. In contrast, the HIPE prepared with added PLA showed no phase separation and remained stable for >1 month.

This diversity in stability could be explained to some extent by the differences in viscosity between the two systems. It has been shown that the stability of an emulsion system increases with an increase in viscosity^10,37^ and the fluoro-emulsion prepared with PLA showed a higher viscosity compared to the HIPE prepared without PLA. Furthermore, interfacial tension can also promote emulsion stability.^38^ It is possible that the introduction of PLA reduces interfacial tension between oil and water, weakens the Ostwald ripening effect and prevents small droplets from coalescing into large droplets.

**Fig. 2:**
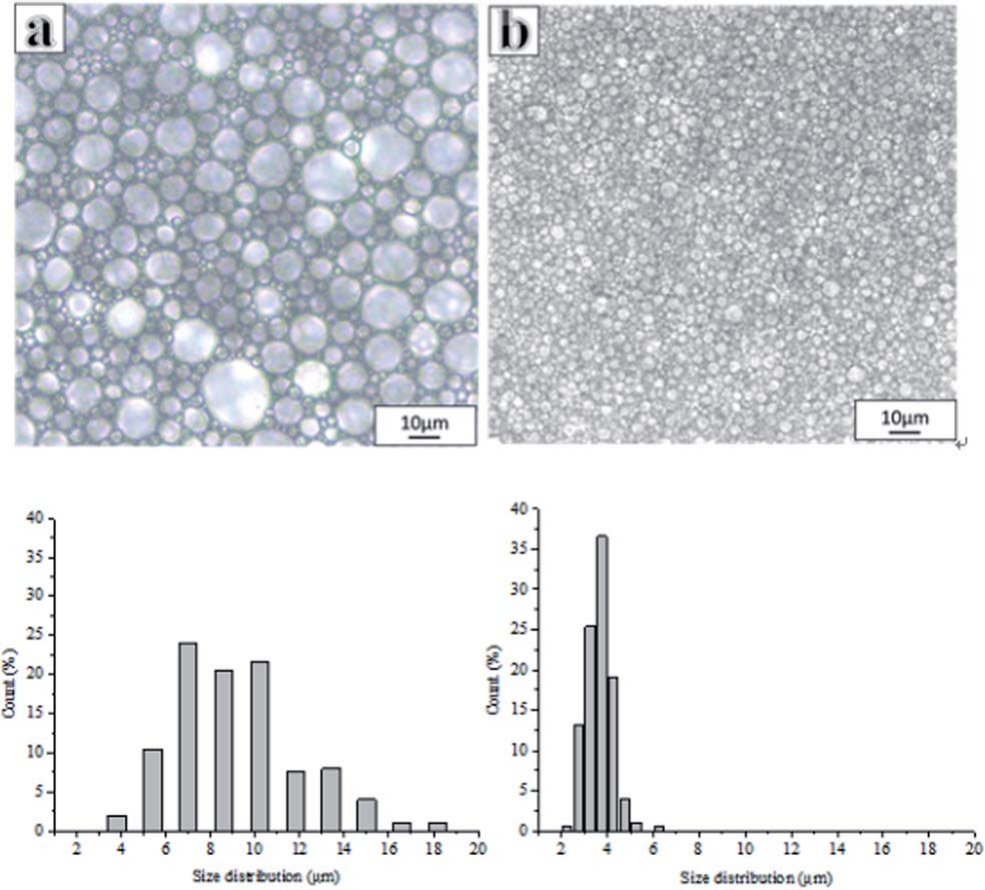
Optical microscope images of emulsion and droplet size distributions (the proportion of smaller pores increased after adding PLA concentration): (a) without PLA, (b) adding PLA (for these samples, Hypermer-B246 was 5 wt% with respect to the total mass of the oil phase).

Fig. 2 shows the optical images and corresponding droplet size distribution graphs of the emulsions prepared with and without PLA. The optical microscopy images (Fig. 2a and b) showed significant differences in droplet sizes, droplet distributions, and a varying thickness of the oil layer between adjacent droplets in both emulsions. The average droplet size of an emulsion decreased from 45.23 μm to 3.17 μm following the addition of PLA. The addition of PLA may reduce droplet aggregation and Ostwald ripening and the small droplets remain stable. For this reason, it can be difficult to obtain a small droplet size from conventional emulsions.

**Fig. 3:**
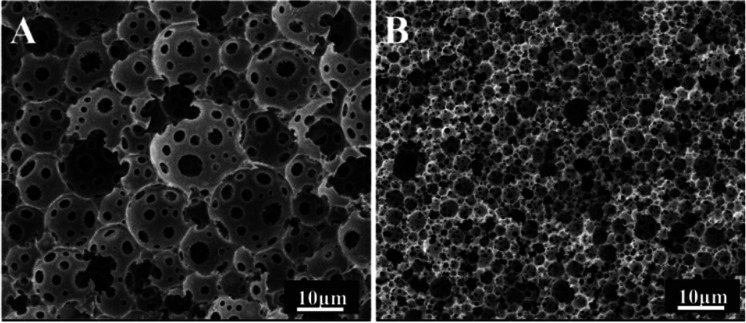
Adjusting the pore morphology by adding PLA, SEM images of the polyHIPEs samples: (A) without PLA, (B) adding PLA (for these samples, Hypermer-B246 was 5 wt% with respect to the total mass of the oil phase, scale bar 10 μm).

Inspection of the morphology of the porous materials post emulsion polymerization, showed that the average pore sizes of polyHIPE prepared with PLA were smaller than those produced without PLA, consistent with the observed (small) emulsion droplets sizes. The porous material prepared without PLA had the large pore structure of a conventional polyHIPE,^1,10,39^ and the average pore diameter was 45.24 μm (Fig. 3A).

Furthermore, the pore size of the emulsion droplets prepared without PLA was smaller (average droplet size of 10 μm) than that of the polymerized material (average pore size 45 μm), which was due to the poor stability of emulsion. When the emulsion was observed using an optical microscope, droplet coalescence was observed (ESI Video S1†). Hence, during polymerization the small droplets may continue to aggregate into larger droplets. The droplet size in the emulsion containing PLA was similar to the pore size of the material after polymerization, indicating that the emulsion with added PLA had good stability (ESI Video S2†).^16,19^

### 4.2 Effect of polylactic PLA

**Fig. 4:**
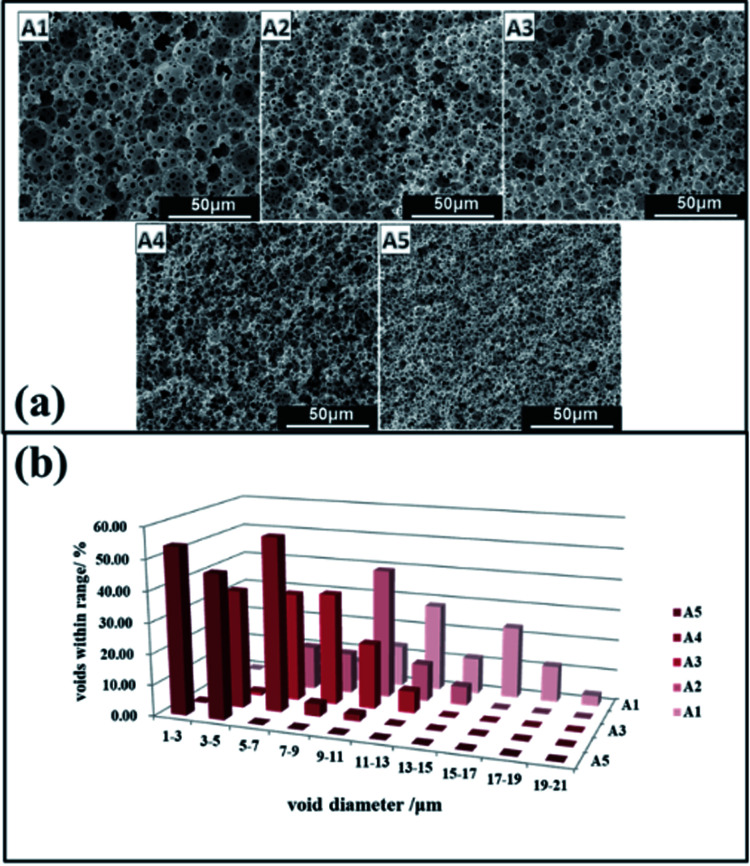
Adjusting the pore morphology by PLA concentration, (a) SEM images of the polyHIPEs (A1) 0 wt%, (A2) 5 wt%, (A3) 10 wt%, (A4) 20 wt% and (A5) 25 wt% of PLA with respect to the continuous phase; (b) pore size distributions.

**Fig. 5:**
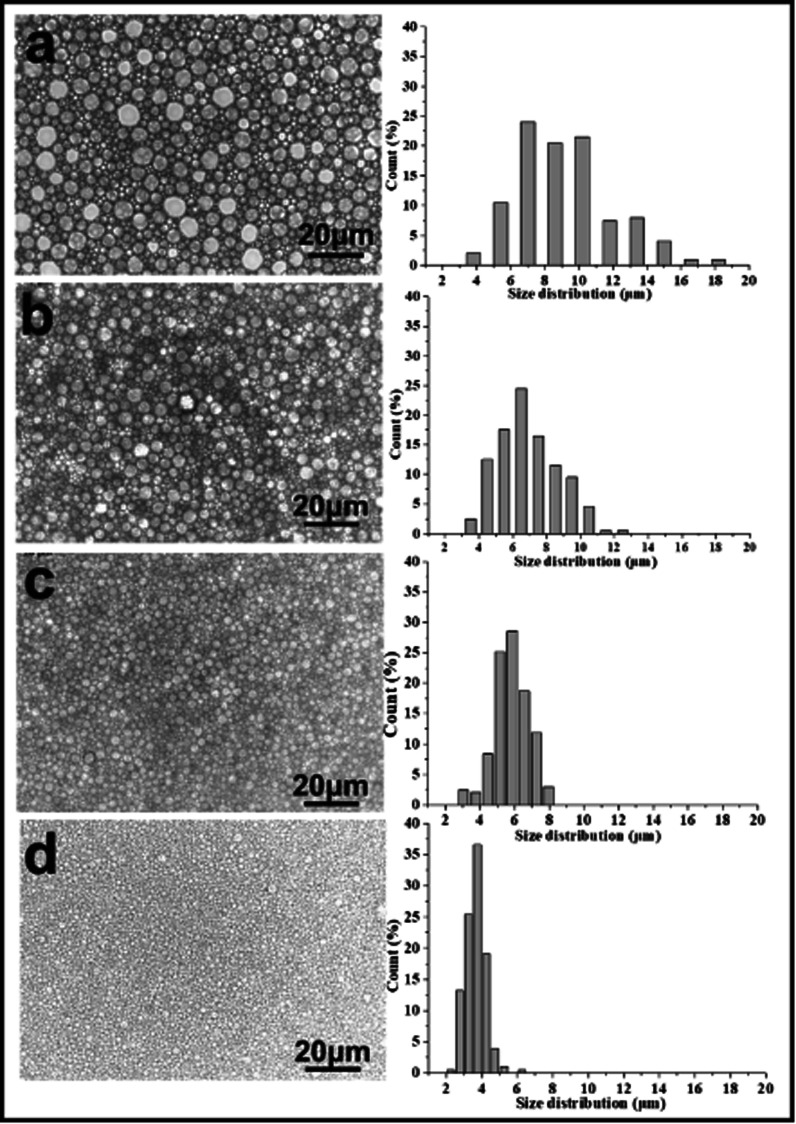
Optical microscope images of emulsion and droplet size distributions (the proportion of smaller pores increased with an increasing PLA concentration): (a) 0 wt%, (b) 5 wt%, (c) 10 wt%, (d) 20 wt% with respect to the continuous phase (for these samples, Hypermer-B246 was 5 wt% with respect to the total mass of the oil phase) (scale bar 20 μm).

A series of emulsion-templated, cross-linked fluorinated materials were synthesized over a range of PLA concentrations (0–20% w/w based on the continuous phase). All other variables (*e.g.* monomer concentration, initiator concentration, surfactant Hypermer-B246 concentration, internal aqueous volume, stirring speed, temperature) were kept constant; Mn of the PLA sample was 11 kg mol^−1^. The results of these experiments are summarized in Table 1 (samples 1–5). In all cases, uniform, white w/o emulsions were observed that filled the entire reaction vessel; these were sufficiently stable to form emulsion-templated polymers that conformed to the internal dimensions of the reaction vessel. Fig. 4 shows a series of electron micrographs and the pore size distribution chart for fluorinated materials synthesized at increasing PLA concentrations. In all cases the familiar macroporous polyHIPE structure, consisting of pores and interconnected windows, was obtained. The median pore diameter measured by SEM corresponded to the average size of the pores connecting the emulsion-templated cells (see Fig. 5).

The images given in Fig. 4 show that as the content of PLA increased, the pore size of the polymerized material decreased from an average pore diameter of 45 μm to 2 μm. Ling *et al.* showed that the pore size of emulsions could be reduced from 32–13 μm by varying the amount of stabilizer.^40^ Zhang and Zhu reported that pore size could be reduced from 110 μm to 53 μm by selection of the appropriate crosslinking agent.^28^ Porous material prepared by the templating method typically has a pore diameter of >5 μm and it is very difficult to obtain smaller pore diameters by this method.^1,9,10,18,19,39^ Consequently, PLA would appear to have a significant role in regulating pore size. The minimum pore size obtained in this study was 1–2 μm: the pore had an even size distribution, they were all interconnected, and they remained intact without collapse and fragmentation.

Further experimental results showed that the pore size could also be altered by changing the molecular weight of the added PLA. The results given in Table 2 show that average pore diameter of microporous polymers was reduced from 7.95 μm to 4.05 μm when the molecular weight of PLA increased from 12 kg mol^−1^ to 100 kg mol^−1^. This effect was relatively small compared to the change in pore size resulting from changes in PLA content.

**Table 2:** Foam average void diameter (*D*_v_), specific surface area (SSA) and foam density (FD) of macroporous polymers

Samples^a^	The molecular weight of PLA	*D* (μm)	SSA (m^2^ g^−1^)	FD (g cm^−3^)
B1	11 kg mol^−1^	7.95 ± 1.32	10.79	0.2078
B2	43 kg mol^−1^	4.95 ± 1.11	15.09	0.1838
B3	70 kg mol^−1^	4.18 ± 0.76	15.27	0.1935
B4	100 kg mol^−1^	4.05 ± 0.45	14.34	0.1885

a For all the samples, the continuous phase of the emulsion consists of a 1 : 1 mixture of TFEMA and DVB, internal phase fraction is 85 wt% with respect to the total emulsion and surfactant Hypermer-B246 is 5 wt% and PLA is 10 wt% with respect to the oil phase containing TFEMA and DVB.

### 4.3 Viscosity of the continuous phase and oil–water interfacial tension

The role of PLA in stabilizing the emulsion can be attributed to the following effects: (1) increased viscosity of the continuous phase, and (2) reduced interfacial tension between w/o. Butler and Hopkinson showed that increasing the aqueous phase (continuous phase viscosity) can help to stabilize the emulsion; a slight increase in the viscosity of the continuous phase increases the shearing force in the mixture, which is responsible for reducing the size of the droplets.^41^

**Fig. 6:**
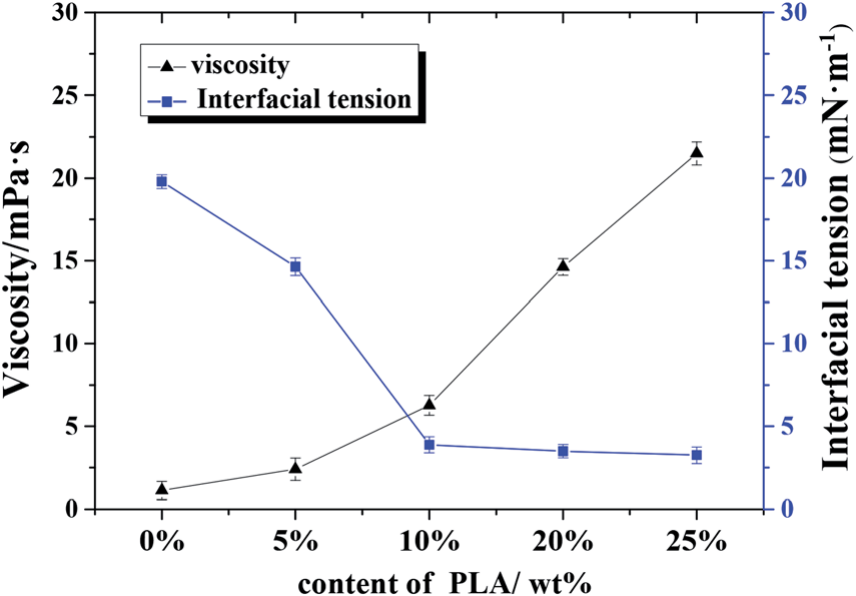
Effect of polylactic acid content on continuous phase viscosity and oil–water interfacial tension.

Because the continuous phase of HIPEs is thinned to films due to the high volume fraction of the dispersed phase, increasing the viscosity of the continuous phase may play an important role in restricting the coalescence of dispersed droplets.^42^ It has been shown that increasing the viscosity of the continuous phase, either by using a more viscous organic liquid (for w/o systems) or increasing the content of crosslinking agent, can give a more stable emulsion.^10^ Similarly, an increase in the viscosity of the emulsion also favors the stability of the emulsion. The viscosity of the continuous phase and the volume fraction of the internal phase both determine the viscosity of the emulsion to some extent. When the internal phase volume fraction is constant, the continuous phase viscosity has the following relationship with the emulsion viscosity:^42^
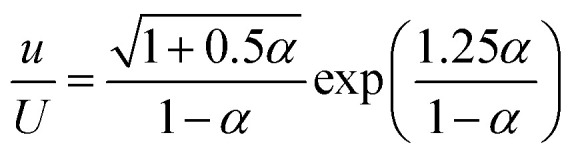
where *u* is the viscosity of the continuous phase, *U* is the viscosity of the emulsion and *α* is internal phase volume fraction. Fig. 6 shows the effect of PLA content on continuous phase viscosity and oil–water interfacial tension. The viscosity of the continuous phase increased significantly with increasing PLA content: an increase in stability of ×20 was obtained when the content of polylactic acid was 25% of the mass of the oil phase; the emulsion with added PLA was sufficiently viscous to maintain its structure after inversion (see ESI Fig. S3†).

To explore the emulsifying ability of PLA, oil phase (4 mL) containing TFEMA (2 mL) and DVB (2 mL), with and without PLA, was stirred for 10 min at 3000 rpm with aqueous calcium chloride solution (4 mL of 0.2 M) without surfactant. Phase separation soon occurred in the samples without PLA, while an even emulsion was formed in the samples with PLA which was sustained for more than 30 min (Nile Red was dissolved in the oil phase as an indicator, see ESI Fig. S4†). To a certain extent, PLA can emulate an emulsifier at the w/o interface due to its amphiphillic structure. HTPB has a similar structure to PLA and its use as a co-stabilizer has also been reported in the literature.^16^ Compared to small molecule emulsifiers, PLA has a high molecular weight (>10 kg mol^−1^) with superior steric hindrance and viscoelastic behaviors, both of which are important for the stabilization of emulsions.

Results from the w/o interfacial tension experiments (see Fig. 6) also support the emulsifier properties of PLA to some extent: The interfacial tension between w/o decreased significantly as the content of PLA increased; the small void size resulted from the decrease in interfacial tension. Hence, the regulation of w/o interfacial tension and continuous phase viscosity is important for the production of stable emulsions.^31^

### 4.4 Mechanical properties

Mechanical properties have always been an important indicator for judging the performance of porous materials. The mechanical properties of all samples were determined. The results showed that the mechanical properties of porous material prepared by surfactant-stabilized emulsion were poor compared to the resulting polyHIPE prepared with PLA. The poor mechanical properties could be attributed to the following characteristics: occasionally, porous materials prepared without PLA cracked during drying or oil adsorption (see ESI Fig. S5†); within the same pore size range, materials with a small pore size controlled by PLA had a complete pore structure and no defects in the pore walls. Porous materials prepared by increasing the surfactant content showed severe shrinkage after drying with loss of pore structure (see ESI Fig. S6†). Hence, increasing the surfactant content to reduce the pore size is not a practical option to improve the performance of porous materials.

Preparation of porous foams with added PLA exhibited enhanced compression resistance compared to porous materials prepared without PLA: compression resistance increased with an increasing content of PLA. The compressive performance of a macroporous polymer is determined by the properties of the materials making up the pore walls.

**Fig. 7:**
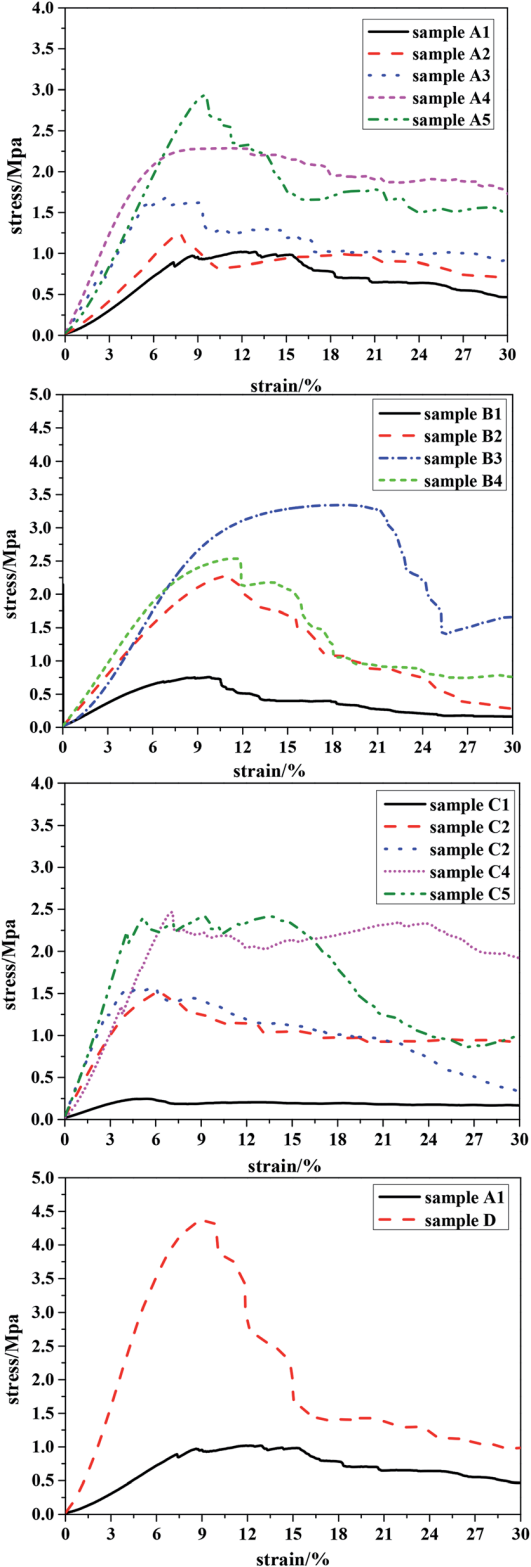
Mechanical performance tests of all the samples. Sample A1–A5 is 0–25 wt% PLA; sample B1–B4 is molecular weight of PLA from 11 kg mol^−1^ to 100 kg mol^−1^; sample C1–C5 is the content of crosslinker 10–90 wt%; sample D is the optimized sample.

A decrease in the pore size of the material results in an increase in the number of walls supported per unit area. Since the pore wall thickness and porosity were similar for all samples (see Table 1), the number of pores per unit volume of the macroporous polymers must be larger for porous polymers with a smaller pore diameter. This leads to the presence of more struts per unit cross-section of the porous polymers with a smaller pore diameter. Therefore, the load required to compress macroporous polymers possessing smaller pores per unit of porous polymer area increases, leading to better compression properties compared to porous polymers with larger pore diameters.^20^ The addition of PLA, with high molecular weight and long polymer chains, most likely increases chain mobility during mechanical deformation, and the recovery processes, to achieve further gains in compressive modulus. The results given in Fig. 7(A1–A5) showed that the amount of PLA had an important effect on the mechanical strength of the polyHIPE: polyHIPEs prepared with a higher PLA content (*e.g.* 25 wt% to oil phase) demonstrated good compressive performance.

When polyHIPE were prepared using a constant concentration of PLA, Fig. 7(B1–B4) shows that the compressive performance increased as the molecular weight of PLA–OH increased: the compression resistance reached an optimum value (2.5 Mpa) at a molecular weight of 70 kg mol^−1^; and then weakened as the molecular weight was increased to 100 kg mol^−1^. The latter observation could be explained by the increased viscosity at the higher molecular weight of PLA–OH. Hence, the prepared emulsion using high molecular weight PLA–OH was too viscous to slip between the emulsions when it was transferred from the agitator to the reactor, resulting in a decrease in the mechanical properties of the porous material.

Rezanavaz and Fee showed that crosslinking agent content and molecular weight could affect the mechanical properties of porous materials.^10^ Furthermore, the mechanical strength of the polyHIPE can be further enhanced by increasing the amount of crosslinking agent (DVB) in the monomer phase of the emulsions before polymerization.^43^

Mechanical performance tests for samples prepared with crosslinking agent in the range 10–90 wt% are shown in Fig. 7(C1–C5): at 10 wt% DVB, the values of crush strength and Young's modulus were extremely low; at 70 wt% DVB, the values of crush strength and Young's modulus increased to 2.48 and 28.87 MPa respectively. This observation could be attributed to the enhancement of the cross-linking density and thus reinforcement of the polymeric framework as the concentration of crosslinking agent increased.

Overall, we found that sample B4 (cross-linking agent content 70 wt%) had better mechanical properties compared to samples prepared at other ratios of TFEMA to DVB. Based on the above research, we elected to prepare a sample that had good compression resistance by optimizing the effects of the three key variables, *i.e.* the concentration of PLA–OH, the molecular weight of PLA–OH and concentration of the cross-linking agent. The compressive properties of the optimized sample are shown in Fig. 7(D).

For the optimized sample (D), the values of crush strength and Young's modulus were >4.2 and >64 MPa, respectively, representing a significant improvement in mechanical properties compared to many engineering foams, such as melamine, polystyrene, and graphite.^20^ For example, the Young's modulus of polystyrene polyHIPEs prepared by the conventional HIPE method was ≤10 MPa.^44–46^

### 4.5 Oil–water separation performance of polyHIPE oil adsorbents

**Fig. 8:**
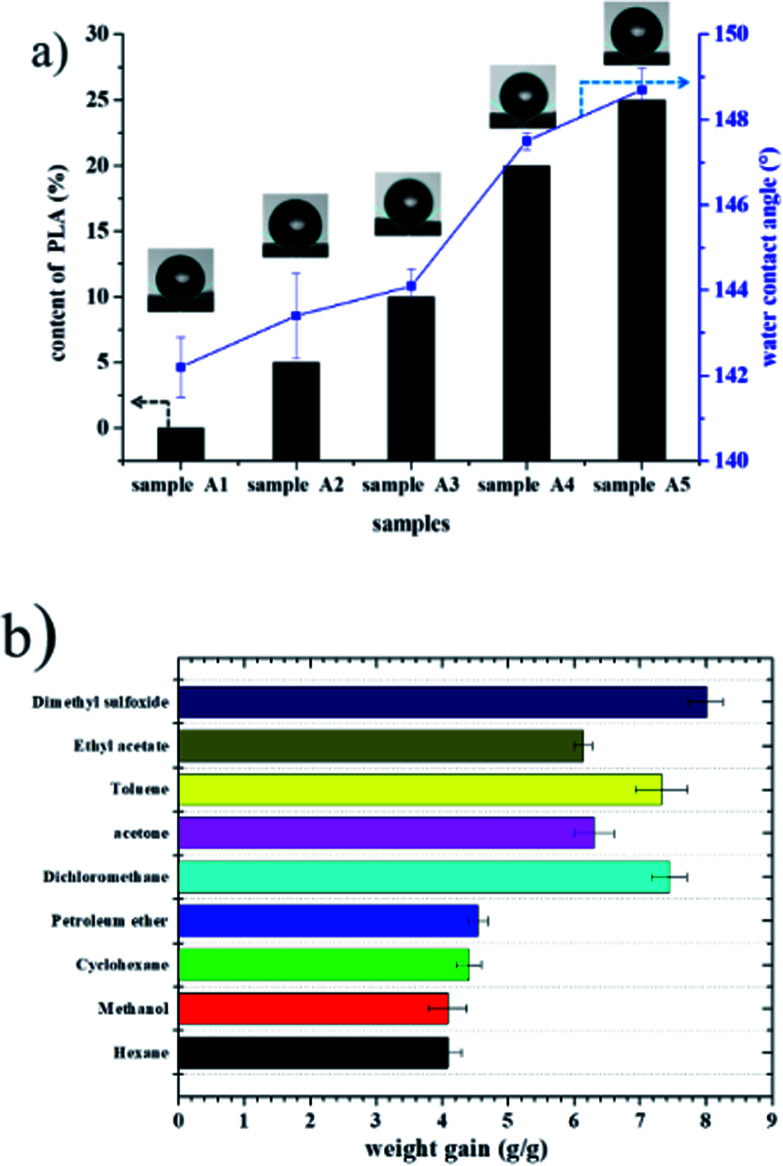
(a) Water contact angle of fluoro-polyHIPEs with different content of PLA; (b) adsorption capacity of the as-prepared polyHIPE (sample A5) for various organic solvents.

Recently, organic solvent spills have received considerable attention due to economic and environmental concerns. Fig. 8 shows the water contact angle and adsorption capacities of polyHIPE. As expected, all the porous materials prepared with PLA exhibited enhanced hydrophobic and oleophilic characteristics. For example, the water contact angle of sample A5 was 148.30° (Fig. 8a), indicating that the sample was hydrophobic. When toluene was dropped on the surface of sample A5, it was adsorbed immediately and caused slight swelling of the sample, confirming its oleophilic property. Because these materials repel water but adsorb oil, monolithic materials have been evaluated for oil adsorption applications such as the separation of oil and organic pollutants from water.^1,18,19^ Hexane, methanol, cyclohexane, petroleum ether, dichloromethane, acetone, toluene, ethyl acetate, and dimethyl sulfoxide were used as target oils to test the adsorption capacities of a polyHIPE foam (sample A5). The adsorption of capacity of foam A5 for nine organic liquids ranged from 4.0–7.4 times its original mass (see in Fig. 8).

## 5. Conclusions

In summary, a fluorinated monolith containing PLA was prepared using the emulsion template method. The addition of PLA stabilized the fluorinated HIPE while the negative effects of surfactants on material properties were largely avoided since the amount of surfactant used was much reduced. The introduction of PLA achieved a significant reduction in the pore size and a corresponding improvement in mechanical properties. A series of fluoroporous polymers were prepared by adjusting several factors including the content of PLA, the molecular weight of PLA, and the ratio of TFEMA to DVB in the oil phase. The mechanical properties of these monoliths were greatly improved compared to previously prepared fluoro-porous materials.

## Conflicts of interest

Author declares no conflict of interest for this research paper.

## Supplementary Material

RA-009-C9RA08226C-s001

RA-009-C9RA08226C-s002

RA-009-C9RA08226C-s003
