# Supplementary material for: A facile fabrication of porous fluoro-polymer with excellent mechanical properties based on high internal phase emulsion templating using PLA as co-stabilizer
Source: RSC Adv. 2019 Dec 6;9(69):40513–22. doi: 10.1039/c9ra08226c (PMC9076259; doi:10.1039/c9ra08226c)
Supplement: RA-009-C9RA08226C-s001 [file RA-009-C9RA08226C-s001.pdf]

Electronic Supplementary Material (ESI) for RSC Advances.
This journal is © The Royal Society of Chemistry 2019

Supporting Information

A facile fabrication of porous fluoro-polymer with excellent mechanical properties based on high internal phase emulsion templating using PLA as co-stabilizer

Yongkang Wang[a,1], Umair Azhar[a,1], Jinxuan He[b], Huiying Chen[a], Jianzhi Zhao[a], Ai-min Pang[b,*], Bing Geng[a,*]

[a] *Shandong Provincial Key Laboratory of Fluorine Chemistry and Chemical Materials, School of Chemistry and Chemical Engineering, University of Jinan, Jinan 250022, China*

[b] *Science and Technology on Aerospace Chemical Power Laboratory, Xiangyang, Hubei 441003, China*

---

*Corresponding authors.

E-mail addresses: hw800008@hotmail.com (J. He), chm_gengb@ujn.edu.cn (B. Geng).

[1]Yongkang Wang and Umair Azhar contributed equally in this work.

Content

Structure and characterization of PLA-OH………………………………………….. S1

Figure S1………………………………………………………………………….....S2

Figure S2………………………………………………………………………….....S2

Figure S3……………………………………………………………………………S3

Figure S4……………………………………………………………………………S3

Figure S5……………………………………………………………………………S3

Figure S6……………………………………………………………………………S4

Figure S7…………………………………………………………………………S4

Structure and characterization of PLA-OH

A one-pot method of lactide-ring opening polymerization was adopted to synthesize PLA-OH polymer.[1] The composition and the structure were confirmed by FT IR，^{1}H NMR and GPC.[2]

The FT-IR spectral frequencies and their assignments characteristic of the polymer structure (Figure S 1a) were as follows: 1757 cm^{-1}, C=O stretching vibration; 1131 cm^{-1} stretching vibration of C-O; 2995 and 2850 cm^{-1}, stretching vibrations of methyl (-CH_3) and methylene (-CH_2-) groups, respectively; 1454 cm^{-1}, bending vibration of (-CH_2-) groups; 3494cm^{-1}, hydroxyl group (–OH).

The structure of PLA-OH was also confirmed by ^{1}H NMR (see Fig.1B **a-d**). The mean degree of polymerization of PLA-OH, calculated from the ratio of the integrated signals at δ 7.1–8.1 (terminal benzene ring -H) and δ 1.5 (–CH_3), ranged from 40 to 300. PLA-OH [($C_6H_5CH_2O$-($OCCH(CH_3)O)_n$-H]: ^{1}H NMR ($CDCl_3$ with 0.05% v/v TMS, 400 MHz): δ 7.1 (**a** 5H), δ5.1 (**c** 1H), δ 1.6 (**d** 3H).

The molecular weight distribution of the di-block copolymer was characterized by GPC. Single peaks were obtained for each GPC chromatogram using different elution solvents (see Fig. 1c) (the differences in retention times are due to the different solvents which are related to molecular weight). Good control of the ring-opening polymerization synthesis was demonstrated for both PLA3000-OH (Đ=1.32 Mn=3000 g/mol) and PLA15000-OH (Đ=1.21, Mn=15000 g/mol).

References

[1] L.T. Lim, R. Auras, M. Rubino, Processing technologies for poly(lactic acid), Progress in Polymer Science 33(8) (2008) 820-852.
[2]M. Seo, M.A. Amendt, M.A. Hillmyer, Cross-Linked Nanoporous Materials from Reactive and Multifunctional Block Polymers, Macromolecules 44(23) (2011) 9310-9318.

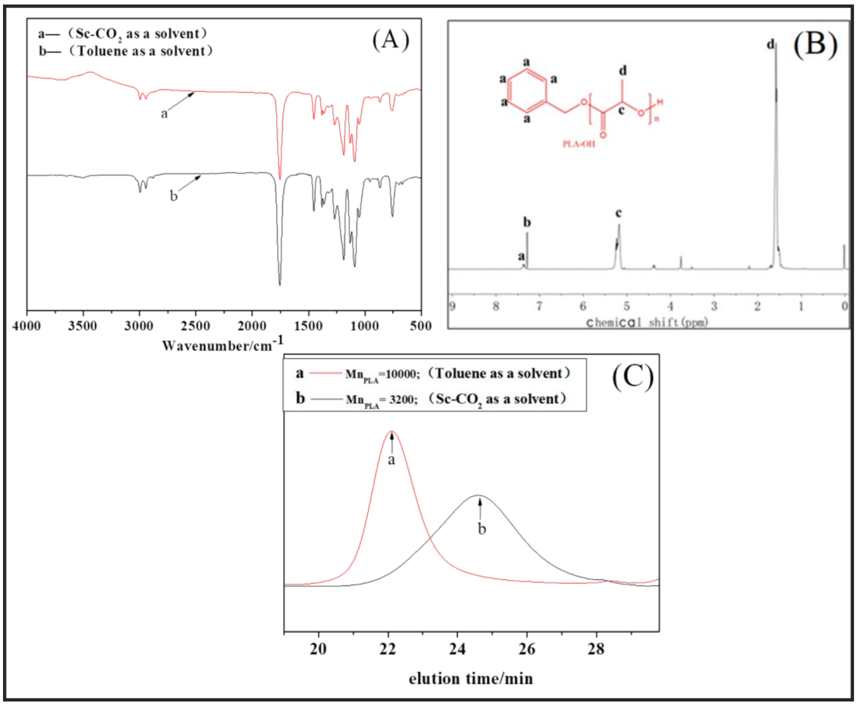

Figure S1 (A) FTIR, (B) ^{1}H NMR and (C) GPC curves of PLA-OH

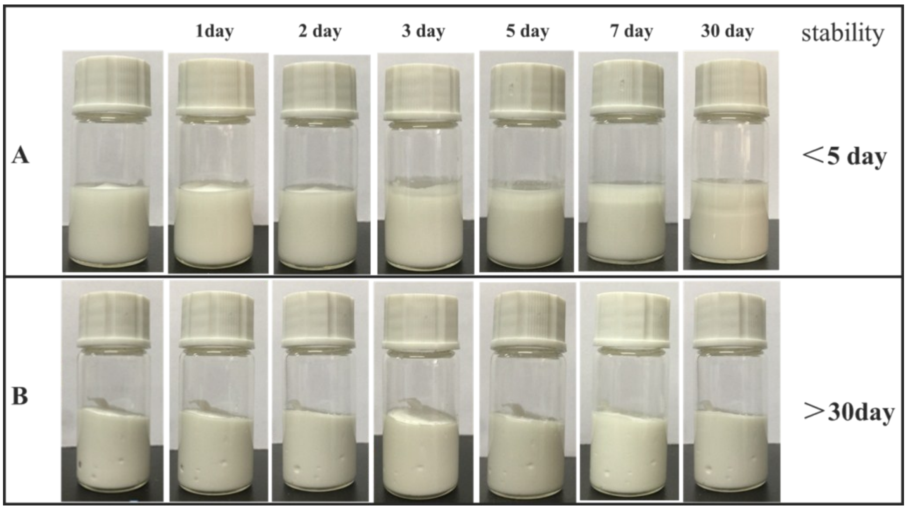

Figure S2 Stability of HIPE under different standing time (A) without PLA, (B) adding PLA.

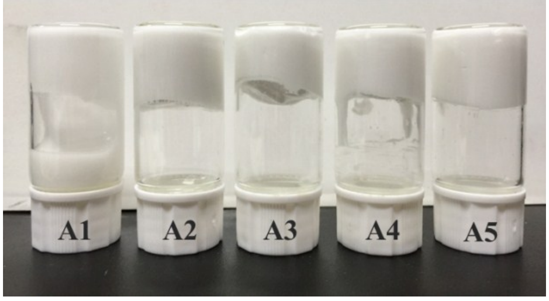

Figure S3 Image of HIPE with (A1) 0 wt%, (A2) 5 wt%,(A3) 10 wt%,(A4) 20 wt%,(A5) 25 wt% PLA with the respect to the oil phase.

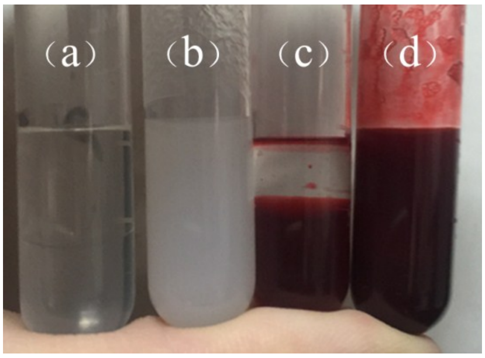

Figure S4 a) only an oil-water mixture, b) an oil-water mixture with polylactic acid, c) an oil-water mixture with an oil-soluble dye in the oil phase, and d) an oil-water mixture with polylactic acid, oil-soluble dye in the oil phase.

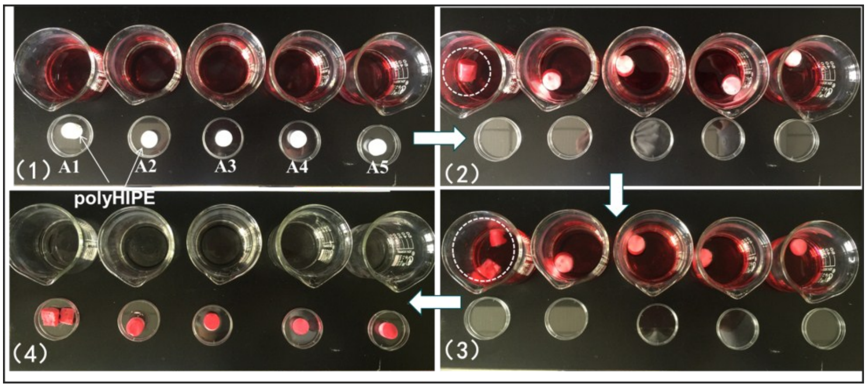

Figure S5 Image of polyHIPE oil absorption test (A1) sample A1 (without PLA) , (A2) sample A2-A5 (with 5wt%-25wt% PLA).

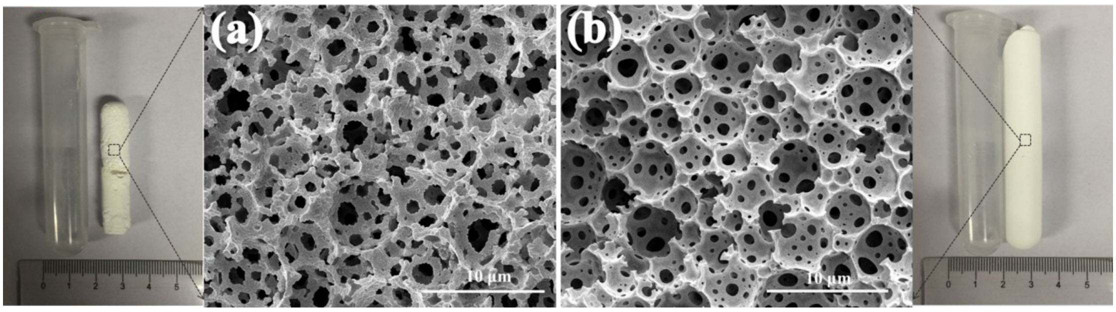

Figure S6 SEM image and morphology of polyHIPE with different concentration of b246 and PLA; a) b246(25wt%), b) b246(3wt%)+PLA(25wt%).
